# The Chemotherapeutic Efficacy of Hyaluronic Acid Coated Polymeric Nanoparticles against Breast Cancer Metastasis in Female NCr-Nu/Nu Nude Mice

**DOI:** 10.3390/polym15020284

**Published:** 2023-01-05

**Authors:** Hassan A. Almoustafa, Mohammed Abdullah Alshawsh, Fouad Saleih R. Al-Suede, Salah Abdulrazak Alshehade, Amin Malik Shah Abdul Majid, Zamri Chik

**Affiliations:** 1 Department of Pharmacology, Faculty of Medicine, Universiti Malaya, Kuala Lumpur 50603, Malaysia; 2 EMAN Biodiscoveries Sdn. Bhd., A1-4, Lot 5, Persiaran 2/1, Kedah Halal Park, Kawasan Perindustrian Sungai Petani, Sungai Petani 08000, Malaysia; 3 Faculty of Bioeconomic & Health Sciences, University Geomatika Malaysia, Kuala Lumpur 54200, Malaysia; 4 ACRF Department of Cancer Biology and Therapeutics, The John Curtin School of Medical Research, Australian National University, 131 Garran Rd., Acton 2601, Australia; 5 Faculty of Medicine, Universiti Malaya Bioequivalence and Testing Centre (UBAT), Universiti Malaya, Kuala Lumpur 50603, Malaysia

**Keywords:** hyaluronic acid, PEG-PLGA nanoparticles, nanoprecipitation, athymic mice, doxorubicin

## Abstract

Polyethylene glycol (PEG) coated Poly lactic-co-glycolic acid (PLGA) nanoparticles (NPs) for cancer treatment are biocompatible, nonimmunogenic and accumulate in tumour sites due to the enhanced permeability and retention (EPR). Doxorubicin (DOX) is a potent but cardiotoxic anticancer agent. Hyaluronic acid (HA) occurs naturally in the extra-cellar matrix and binds to CD44 receptors which are overexpressed in cancer metastasis, proven to be characteristic of cancer stem cells and responsible for multidrug resistance. In this study, an athymic mice model of breast cancer metastasis was developed using red fluorescent protein (RFP)-labelled triple negative cancer cells. The animals were divided into four treatment groups (Control, HA-PEG-PLGA nanoparticles, PEG-PLGA nanoparticles, and Free DOX). The tumour size growth was assessed until day 25 when animals were sacrificed. Mice treated with HA-PEG-PLGA NPs inhibited tumour growth. The tumour growth at day 25 (118% ± 13.0) was significantly (*p* < 0.05) less than PEG-PLGA NPs (376% ± 590 and control (826% ± 970). Fluorescent microscopy revealed that HA-PEG-PLGA NPs had significantly (*p* < 0.05) less metastasis in liver, spleen, colon, and lungs as compared to control and to Free DOX groups. The efficacy of HA-PEG-PLGA NPs was proven in vivo. Further pharmacokinetic and toxicity studies are required for this formulation to be ready for clinical research.

## 1. Introduction

Failure in clinical trials after positive in vitro-in vivo results [1,2,3] has become a great challenge in nanomedicine. New technologies are constantly being proposed by researchers to consider the complexity of human physiology, leading to a better clinical translation of research outcomes [4]. Poly lactic-co-glycolic acid (PLGA) polymer nanoparticles (NP) are among the most important candidates for the near future development of cancer nanomedicine [5].

PLGA nanoparticulate systems are prone to be uptaken by the reticuloendothelial cells in the liver, after being opsonized by the compliment enzymes due to their hydrophobic characteristics. To overcome this obstacle, the surface of the nanoparticles is coated with a hydrophilic compound. The most important among these compounds is PEG [6], which helps the nanoparticles to target tumours passively as their size and surface hydrophilicity favors their leak into tumor pores ranging in size between 100–3000 nm [7,8]. The impaired lymph vessels of the tumour then prevent the nanoparticles from leaking out of the tumour microenvironment. This phenomenon is called the enhanced permeability and retention (EPR) or passive targeting [9].

Active targeting on the other hand utilises the affinity of one or more of the components of the nanoparticulate system to certain target parts of the tumour, whether it is the extracellular matrix, the vasculature, the surface, or other different parts of the cancer cells. In these targeting techniques, hyaluronic acid (HA) which binds to C44 receptors has gained significant interest in the last decade [10].

When nanoparticles are formed from amphiphilic block copolymers such as PEG-PLGA, nanoprecipitation becomes a favorable method as it reduces or eliminates the need for a surfactant and simplifies the preparation method [11]. It is also particularly useful with highly potent drugs such as Doxorubicin, which is an excellent model for anticancer research because of its high toxicity and its well-studied pharmacodynamic and pharmacokinetic behaviors. However, its low logP puts it at a disadvantage when using nanoprecipitation. To counter that while keeping the simplicity of the formulation, pH can be increased [12] and the compound needs to be released from its salt form [13]. Anthracyclines, however, are considered ideal hydrophilic candidates for nanoprecipitation as their high potency can overcome the problem with the low concentration of the formulation [11].

This work represents in vivo efficacy testing on a previously developed and reported cancer metastasis targeted nanoparticle formulation proven to significantly decrease resistance to doxorubicin in hypoxia stressed breast cancer metastatic cells.

## 2. Materials and Methods

### 2.1. Materials

PLGA with 6kDa MW was purchased from LACTEL^®^ (Hoover, AL, USA) and Methoxy polyethylene glycol (MPEG) (MW 2000Da) and diamine polyethylene glycol (PEG) (MW 2000Da) were purchased from Laysanbio^®^ (Arab, AL, USA). Hyaluronic acid (MW 1.38 mDa) was obtained from Lifecore^®^ Biomedical (Chaska, MI, USA). 1-Ethyl-3-(3-dimethylaminopropyl) carbodiimide (EDC) and *N*-hydroxysuccinimide (NHS) were purchased from Sigma (St. Louis, MO, USA). Doxorubicin HCl (DOX) was from BOC Sciences (Upton, NY, USA). Ultracentrifuge tubes and slides were obtained from Pall (Port Washington, NY, USA). Slide-A-Lyzer Dialysis disks (MWCO 3000kDa) from Fisher Scientific (Waltham, MA, USA), while the MDA-MB-231/RFP (tagged with red fluorescent protein) triple negative breast cancer cell-line was purchased from Cell Biolabs Inc. (San Diego, CA, USA). Other chemicals include acetate buffer, triethylamine, Phosphate-Buffered Saline, and analytical grade solvents.

### 2.2. Nanoparticle Preparation

#### 2.2.1. Preparation of Block Copolymer

The preparation method was detailed in our previous publication [14] using the carbodiimide reaction. Hence, 60–200 mg of PLGA were dissolved in 1-2 mL acetonitrile. Then, 5 times molar excess of EDC and NHS were added and stirred for two hours. The activated PLGA was then precipitated and washed three times with excess of methyl alcohol and centrifuged at 2500 g for 10 min. The activated PLGA-NHS was then dried by nitrogen and redissolved in acetonitrile. After that, a 3-fold molar excess of PEG (MPEG for untargeted nanoparticles and diamine PEG for nanoparticles coated with HA) was added and stirred for 24 h. The resulting copolymer was then spun at 5000 g with 10 times the volume of the reaction solution of methanol. To get rid of the excess non-reacted PEG, the process was repeated three times. To prepare the copolymer for use later, it was dried under nitrogen and kept at −20 °C.

#### 2.2.2. Preparation of NPs

A modified nanoprecipitation was used to create the nanoparticles [15]. In short, 100 L of DMSO was used to dissolve 2 mg of doxorubicin HCl, which was subsequently desalted for three hours while being stirred using a 2× molar ratio of TEA buffer [16]. The mixture was then diluted to 1 mL using acetone. The dried PEG-PLGA copolymer was added in amounts of approximately 10 mg and mixed completely until dissolved. Under a chemical hood, 9 mL of pH-adjusted PBS solution was rapidly stirred before the organic phase was added slowly and dropwise with a 100 L micropipette. Utilizing ice bags to maintain a cold temperature, stirring continued for eight hours to completely remove the acetone. To remove the DMSO, Slide-A-Lyzer-G2-Dialysis-Cassettes were used, and the dialysis was performed over 48 h with water replaced at 3, 6, 12, 24, and 36 h.

#### 2.2.3. Hyaluronic Acid Coating

High MW HA-Na (*HANa_weight_ = 0.1 × PEGPLGA_weight_*) was dissolved in sodium acetate buffer for 3 h and pH was adjusted to 4.5. Then, HA was activated using 5× molar ratio of EDC and NHS for 2 h. The activated HA-NHS was then stirred with PEG-PLGA NPs for one day before the targeted NPs solution was ready [17].

#### 2.2.4. Nanoparticle Characterization

Nanoparticles were checked for chemical composition using FTIR and NMR, size, surface charge, encapsulation efficiency, and morphology and the results were detailed in our previous publication [14].

#### 2.2.5. In Vivo Athymic Nude Mice Study

**Figure 1:**
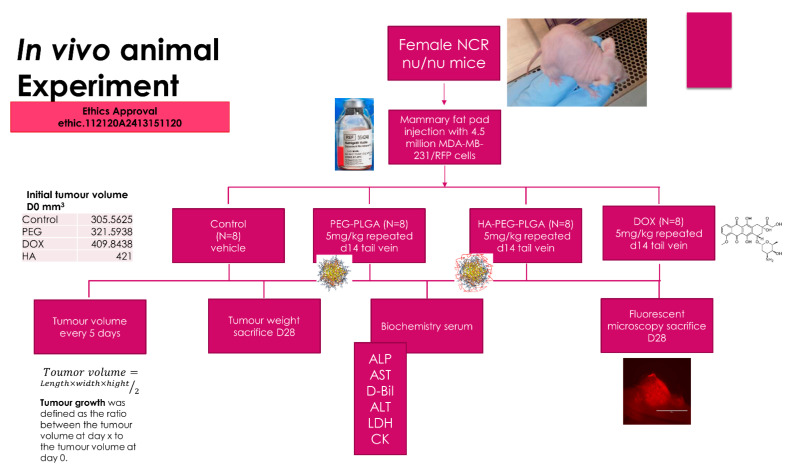
Experimental design of the in vivo experiment (DOX: free doxorubicin, PEG-PLGA: Doxorubicin loaded PEG-PLGA nanoparticles, HA-PEG-PLGA: Hyaluronic acid coated Doxorubicin loaded PEG-PLGA nanoparticles).

Animal ethics approval was obtained from The Animal Ethics Committee, Malaysia Division of Eman Research Ltd. (Ethic No. 112120A2413151120). Athymic NCr female nu/nu nude mice (Taconic Farms Inc., USA) were used in this experiment. Figure 1 shows the experimental design of this study.

#### 2.2.6. Drug Dose Toxicity Test

Three animals per group were used for this experiment. Each was injected with 25 mg/kg of free doxorubicin (DOX), Doxorubicin loaded PEG-PLGA nanoparticles (PEG-PLGA), or Hyaluronic acid coated Doxorubicin loaded PEG-PLGA nanoparticles (HA-PEG-PLGA). The experiment was repeated after two weeks. Animal were observed for their weight as well as food and water intake measured once every week to ensure no significant changes in their weight compared to the growth chart provided by the company [18].

#### 2.2.7. Animal Model Establishment

An animal breast cancer model was established by using MDA-MB-231/RFP cells which were injected into nude mice at a concentration of 45 × 10^5^ cells in 200 µL of cell culture media and 200 µL of Matrigel matrix into the right lower abdomen mammary fat pad of 50 mice. At day 21 from injection, only 32 mice developed a visible tumour and were divided into 4 groups (Control, DOX, PEG-PLGA, HA-PEG-PLGA). Two animals from each group died before the end of the experiment.

#### 2.2.8. Treatment

After 21 days of the tumour induction, animals were randomly assigned into four groups (*n* = 8). The groups were injected via tail vein with 200 µL of normal saline (control), 5 mg/kg DOX, 5 mg/kg PEG-PLGA, or 5 mg/kg HA-PEG-PLGA. The dose was repeated after two weeks.

#### 2.2.9. Tumor Size Measurement

Tumor size was measured manually using a manual caliper, and the tumour volume was calculated using the following equation:

(1) Tumor volume=Length×width×height2

Tumour growth was defined as the ratio between the tumour size at day X to the tumour size at day 0 when the treatment started.

#### 2.2.10. Fluorescent Microscopy

Harvested organs (liver, spleen, lungs, and colon) were examined under florescent microscopy (EVOS FI Fluorescence microscope AMG, Bothell, WA, USA) with FITC filter to look for the fluorescent emission of the MDA-MB-231/RFP cells. When observed, the organ is considered bearing a metastatic lesion.

## 3. Results

### 3.1. Nanoparticle Formulation and Characterization

The chemical composition of the nanoparticles was confirmed, and they had an average size of 106 ± 5 nm, a negative surface charge (−15 ± 3 mV), and encapsulation efficiency of 73.3 ± 4.1% [14].

### 3.2. Drug Dose Toxicity Test

**Figure 2:**
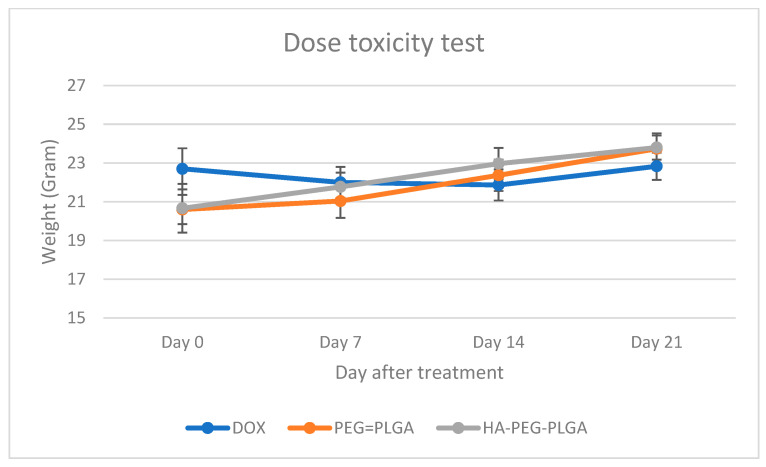
Change in weight of healthy animals after being injected with 25 mg/kg of (DOX: free doxorubicin, PEG-PLGA: Doxorubicin loaded PEG-PLGA nanoparticles, HA-PEG-PLGA: Hyaluronic acid coated Doxorubicin loaded PEG-PLGA nanoparticles).

No noticeable change in animal activity was visually observed and while animal weight dropped when treated with free DOX, animals treated with both nanoparticle formulations continued to increase in weight (Figure 2).

### 3.3. Tumour Growth

**Figure 3:**
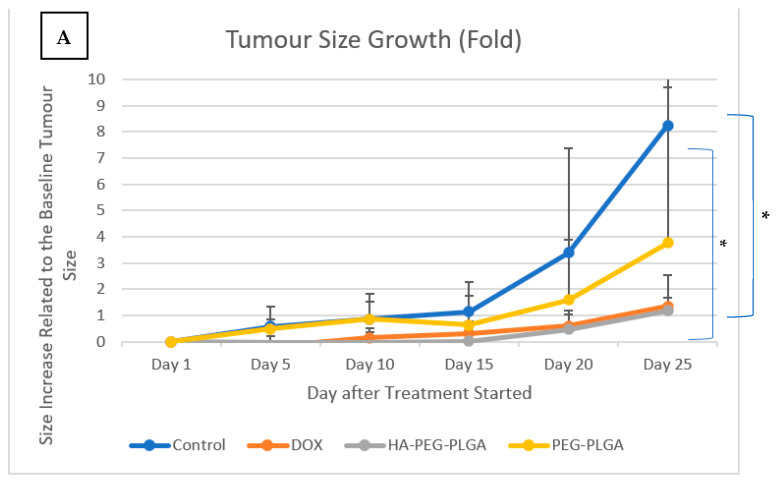

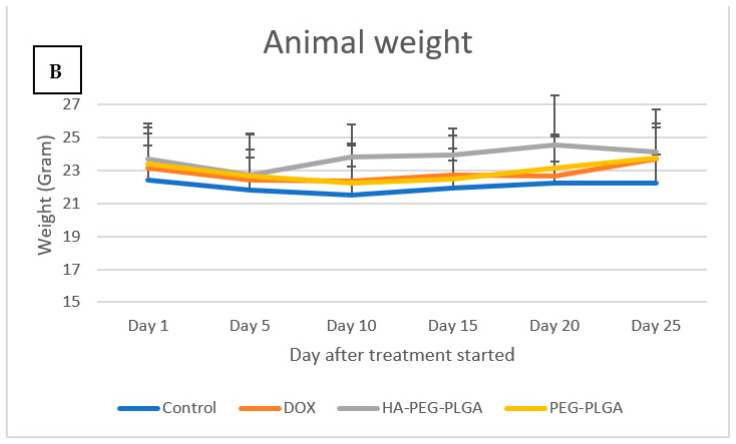
(**A**): Primary tumour growth results presented in fold increase relative to day 1. * Indicates *p* ≤ 0.05 compared to control, (**B**): Animal weight (DOX: free doxorubicin, PEG-PLGA: Doxorubicin loaded PEG-PLGA nanoparticles, HA-PEG-PLGA: Hyaluronic acid coated Doxorubicin loaded PEG-PLGA nanoparticles).

**Figure 4:**
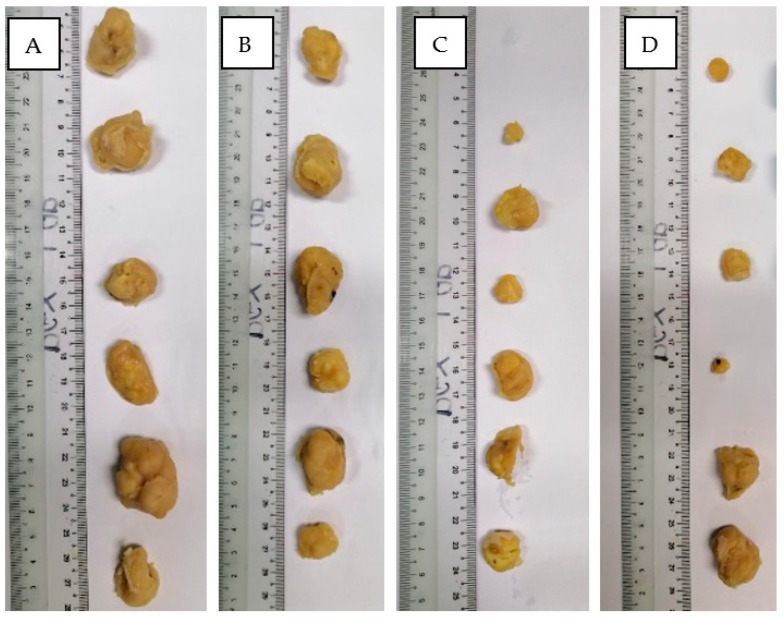
Harvested tumours of (**A**) control (**B**) DOX (**C**) PEG-PLGA (**D**) HA-PEG-PLGA groups.

From eight mice per group, two mice died before Day 25 in each group. Only six tumors were harvested per group of animals. Figure 3A shows the difference in tumor growth between four groups of animals, where the control group showed an average 8.25 times increase in tumor size, DOX treated group had 1.33 times increase, PEG nontargeted NPs group had an increase of 3.75 times, while HA targeted NPs treated group had a 1.18 times increase in size at Day 25, respectively. Figure 3B shows the change in body weight for the animals. Images of tumours harvested from the four groups at day 28 after anesthesia are combined in Figure 4.

### 3.4. Fluorescent Microscopy

**Figure 5:**
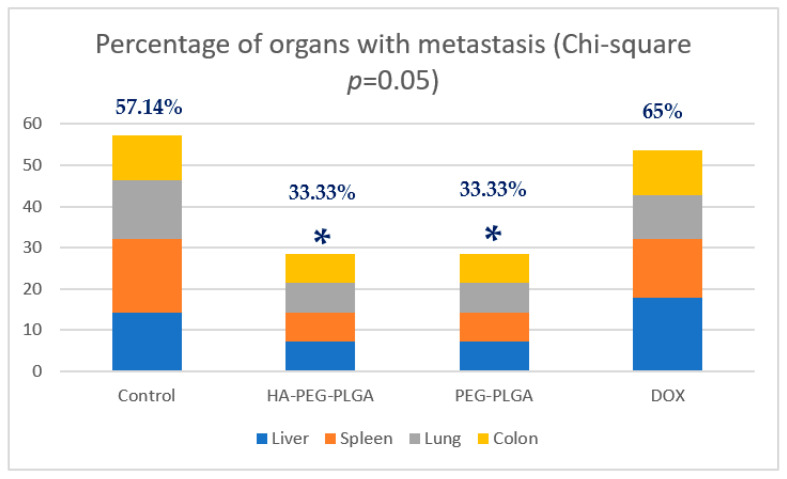
Percentage of organs collected from each group with metastasis detected under fluorescent microscope (* *p* ≤ 0.05 compared to control group) (DOX: free doxorubicin, PEG-PLGA: Doxorubicin loaded PEG-PLGA nanoparticles, HA-PEG-PLGA: Hyaluronic acid coated Doxorubicin loaded PEG-PLGA nanoparticles).

**Figure 6:**
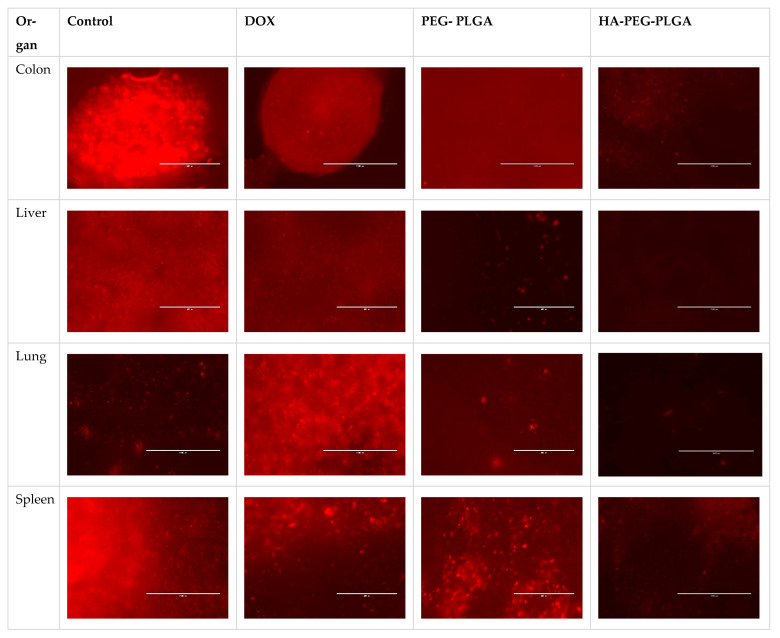
Comparison of the metastatic lesions found in the PEG-PLGA DOX group and the HA-PEG-PLGA- DOX group using fluorescent microscopy.

Metastatic cells were found in four livers of the control group, five of free DOX- treated mice, and only two in HA targeted NPs and PEG non targeted NPs treated groups. Similar results from other harvested organs (spleen, lungs, and colon) with 16 out of 28 (57.14%) harvested organs in control group had metastatic lesions, while eight out of 24 (33.33%) harvested organs in HA- and PEG-treated groups acquired metastatic cells and 15 out of 23 (65%) harvested organs from free DOX group had metastasis (Figure 5). These results were analyzed using Chi square and found to be significant (*p* = 0.05). Figure 6 compares examples of images taken from PEG-PLGA group and HA-PEG-PLGA group.

### 3.5. Biochemistry

For biochemistry parameters, no significant changes were observed between control, DOX, PEG-PLGA, and PEG-PLGA-HA except the alkaline phosphatase (ALP) (Table 1). ALP was elevated significantly *p* < 0.05 in the HA-treated group as compared to control. Free DOX treated mice showed higher cardiac enzymes (CK and LDH) as compared to control. However, the difference was not significant. Mice treated with HA targeted and non-targeted NPs showed lower CK and LDH enzymes as compared to free DOX, which indicates less cardiotoxicity.

## 4. Discussion

In this study, we used nanoprecipitation method to prepare PEG-PLGA-HA DOX and the tumour volume demonstrated similar results, as reported by Tsai et al. [19], where the tumor volume was seven times smaller in the targeted nanoparticles group compared to control. They also used nanoprecipitation to prepare poly (HEMA-co-histidine)-g-PLA and diblock PEG-PLA nanoparticles with folate as a targeting moiety. However, the simplicity in formulation, compared to their formulation that needed two block copolymers, the reduction in particle size by half, along with the cancer stem cells targeting rather than the targeting of folate towards fast dividing and less drug resistant cells are added advantages of our formulation. 

On the other hand, Lu et al. [20] used hollow poly(*N*-vinylimidazole-co-Nvinylpyrrolidone)-g-poly(d,l-lactide) graft copolymers and methoxyl/functionalized-PEG-PLA diblock copolymer NPs on HeLa bearing mice and their findings are comparable to our results in terms of tumour size reduction.

Triple negative breast cancer is defined as the type of breast cancer where cells do not express estrogen, progesterone or HER2 receptors. Thus, it is not affected with treatments that target these types of receptors and exhibits bad prognoses [21]. HA-CD44 targeted nanoparticles are being investigated against this subtype of breast cancer [22,23,24] as it is not responsive to other common receptors and effective current treatments, though generalizing results to other subtypes remains logical and CD44 is expressed in various subtypes of breast cancer that are drug resistant.

Several animal models are proposed for breast cancer. However, when it comes to cancer metastasis and secondary tumour monitoring, most of these models are insufficient [25] and lack enough focus on the metastatic process. While there is no ideal model for human breast cancer or breast cancer metastasis, choosing immunocompromised athymic mice has the advantages of being fast and affordable. In addition, they relatively mimic human tumor development, have easily measured/resected tumors, and have an appropriate tumor microenvironment [26].

One of the simple methods to overcome the limitations of mice models of metastasis is to use fluorescent protein labelled cell lines in immunosuppressed nude mice, which can be tracked with relative ease and high precision as well as allow us to monitor the metastatic progression at different regions [27]. It is important to note that the growth of the breast cancer cells in the animal model may require continuous supply of sexual hormones depending on the type of breast cancer cell line. Choosing a triple negative cell line, such as MDA-MB-231 cells, would eliminate this confounder factor, which adds to advantages of this cell line. In this study, the nontargeted nanoparticles did not reduce the tumour size as compared to the free doxorubicin and targeted nanoparticles This trend was also reported elsewhere [28]. However, the nontargeted nanoparticles can still be considered efficacious based on the organ metastasis study, which showed that the intensity of metastatic lesions and the number of organs involved in metastasis are also lower for targeted nanoparticle as compared to free dox.

It is important to note that comparing the results of such studies is extremely difficult due to the wide variation of animal models used and the variety of cancer diseases chosen to test the complex nano formulations.

The result from tumour metastasis study showed that there was a significant difference between HA-treated group VS. control and no significant difference between HA-treated group vs. free DOX. However, when we take into consideration the result on the tumor size, our formulation can be considered superior to both free DOX (in the tumour size reduction) and PEGylated NPs (in the metastasis reduction).

There are also no significant differences in all serum biochemistry profiles between all treatments showing the safety profiles of all the formulations. However, there is a significant increase in alkaline phosphatase for the HA-PEG-PLGA DOX treated group which can be related to the liver or other tissue damage [29]. However, in a study published by [30], its level was reported to be decreased with HA supplementation. This contradictive result is of value for further investigation of our formulation in term of its toxicity profiles to various organ especially the liver. Cardiac markers CK and LDH were higher in the mice treated with free DOX as compared to untreated mice control. This indicates cardiotoxicity, with a reported adverse effect of DOX. However, the cardiac enzymes were lower in the mice treated with the targeted NPs and this suggests that our formulation has less cardiotoxicity as compared to free DOX. 

## 5. Conclusions

The prepared targeted NPs formulation, HA-PEG-PLGA was proven in this study, to significantly reduce the tumor size in an animal model by half as compared to the PEG-PLGA, and more interestingly it reduces metastasis by half as compared to free DOX. Although the number of organs having metastasis appeared to be the same in targeted and non-targeted groups, the photos clearly show that the amount of metastasis is less in the targeted HA-PEG-PLGA group. Using hyaluronic acid as targeting moiety (targeting CD44 overexpressing cancer cells) makes it superior to both conventional therapy and stealth-technology based NPs, which are currently available in the market.

More research should be conducted to investigate the molecular level of the particle’s tumour interactions, such as measurement of targeting affinity, which can help in optimizing the HA molecular weight to be used. Further investigation is proposed to establish the pharmacokinetics and toxicity profile, focusing on the cardiotoxicity of the formulation, and histologic analysis can be useful to detect other signs of toxicity and in assessing the metastatic lesions in order to move forward to successful clinical trials and commercialization.

## Figures and Tables

**Table 1:** Serum biochemistry results obtained at the end of experiment for all treated mice.

Sample	ALP (U/L)	AST (U/L)	CK (U/L)	DBil (μmol/L)	ALT (U/L)	LDH (U/L)
Control	68.66 ± 14.9	219.5 ± 78.68	388.83 ± 317.24	0.5 ± 0.17	45 ± 25.62	1581.6 ± 1309.6
HA-PEG-PLGA	142.5 ± 48.8 *	221.5 ± 74.10	612.5 ± 442.9	0.56 ± 0.11	57.25 ± 12.41	1343.5 ± 879.3
PEG -PLGA	118 ± 51.1	159.5 ± 27.6	253.5 ± 125.1	0.65 ± 0.12	42.5 ± 11.5	710 ± 411.4
DOX	93.8 ± 25.3	293.8 ± 94.4	854.6 ± 1114.7	1.75 ± 1.9	34 ± 13.28	1890.2 ± 833.9

Data are presented as Mean ± SD, * *p* < 0.05 compared to control.

## Data Availability

Not applicable.
